# Ankle Exoskeleton Assistance Increases Six-Minute Walk Test Performance in Cerebral Palsy

**DOI:** 10.1109/OJEMB.2021.3135826

**Published:** 2021-12-15

**Authors:** Benjamin C. Conner, Greg Orekhov, Zachary F. Lerner

**Affiliations:** ^1^ College of Medicine–PhoenixUniversity of Arizona 42283 Phoenix AZ 85004 USA; ^2^ Department of Mechanical EngineeringNorthern Arizona University 3356 Flagstaff AZ 15600 USA

**Keywords:** Ankle assistance, cerebral palsy, exoskeleton, six-minute walk test

## Abstract

Objective: To determine the effects of providing battery-powered ankle dorsiflexor and plantar flexor exoskeleton assistance on six-minute walk test performance and efficiency in children and young adults with cerebral palsy by comparing distance walked under exoskeleton assisted (Assisted) and no device (Shod) walking conditions, and explore the acclimation rate to maximal walking with ankle exoskeleton assistance. Results: Six-minute walk test performance significantly improved under the final Assisted condition test compared to the Shod condition (42 ± 27 m, p = 0.02), surpassing the minimum clinically important difference range for children and young adults with CP. There was no difference in walking efficiency (-0.06 ± 0.1, p = 0.3). Participants had an average acclimation rate of 19.6 m per session. Conclusions: Powered ankle assistance can significantly improve six-minute walk test performance in individuals with mild-to-moderate gait impairment from CP, supporting the use of this intervention to improve functional mobility and walking capacity in this patient population.

## I. Introduction

Cerebral palsy (CP), a pediatric onset movement disorder characterized by slow and inefficient gait patterns [1], is often associated with poor neuromuscular control at the ankle joint [2], [3]. These pathological gait patterns create a significant barrier to physical activity, which is likely detrimental to healthy childhood development [4]. In an effort to lower the barrier to physical activity, wearable powered exoskeleton devices have sought to increase walking speed and efficiency of individuals with gait impairment, including CP [5]. No study to date, including our own, has clinically evaluated the ability of battery-powered robotic assistance to improve aerobic capacity and endurance during high-intensity long-distance walking in children or young adults with CP. The six-minute walk test (6MWT) is a common and reliable clinical assessment of functional exercise capacity; performance on this test is significantly associated with physical activity levels in individuals with CP [6].

The primary aim of this study was to determine the effect of providing ankle plantar flexor and dorsiflexor assistance from a lightweight untethered exoskeleton (Fig. 2(B)) on 6MWT performance in children and young adults with CP. We hypothesized that participants with CP would walk significantly further with exoskeleton assistance compared to no assistance following acclimation. Our secondary aims were to explore the difference in energetic efficiency between the assisted and unassisted conditions, and study the acclimation rate of exoskeleton-assisted 6MWT distance.

## II. Results

**Figure. 1.**
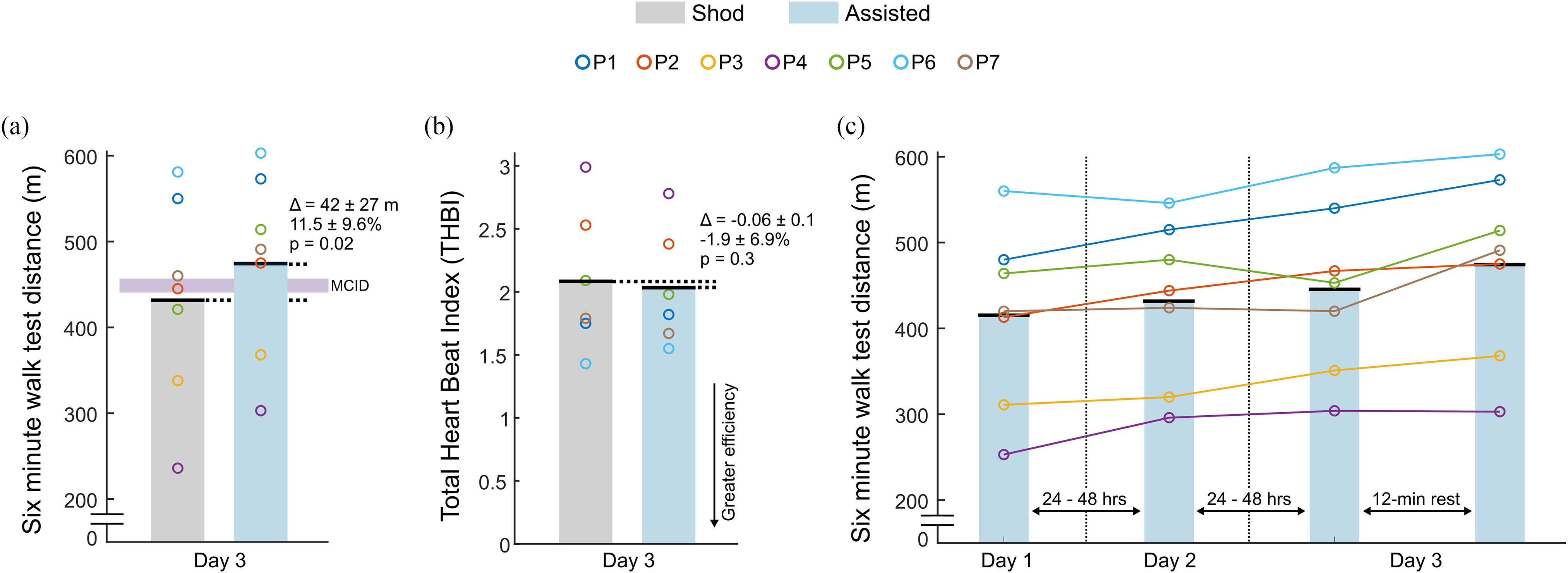
(a) Six-minute walk test performance, where the purple horizontal bar indicates the minimum clinically important difference (MCID) range (4–28 m) for individuals with GMFCS levels I–II, which encompasses the range for individuals with GMFCS level III (9–19 m); (b) Total Heart Beat Index (THBI) under Shod (gray) and Assisted (blue) conditions; and (c) Six-minute walk test performance across exoskeleton acclimation visits, whereby the final Day 3 test was used for comparison to the Shod condition; note that THBI was not measured for P3 due to failure of the heart rate monitor to stay on the participant.

Seven individuals with CP (Table I) completed the three-day protocol without any adverse events. Participants walked significantly further during their final Assisted test compared to their Shod test (42 ± 27 m, p = 0.02; Fig. 1(A)); all participants met or surpassed the minimum clinically important difference (MCID) ranges for CP (GMFCS levels I & II: 4-28 m; GMFCS level III: 9-19 m) (Table I) [7]. There was no significant difference in energetic efficiency between the two conditions (-0.06 ± 0.1, p = 0.3; Fig. 1(B)). Linear regression results indicated a 19.6 m (p = 0.01; Fig. 1(C)) increase in distance walked, on average, across the Assisted tests, producing the following fitted model: 6MWT distance = 19.6 m*(# Assisted tests) + 393 m.

**TABLE I:** Participant Characteristics and Six Minute Walk Test Results

	Gender	Age (y)	Height (cm)	Weight (kg)	GMFCS^a^	CP Type^b^	Regular Assistive Device Use^c^	Δ 6MWT Distance with Exo Assistance	MCID^d^
P1	M	13	148	38.6	II	SD	Bilateral solid AFOs	23 m	4 – 28 m
P2	M	15	158	59.4	II	SD	Bilateral solid AFOs	30 m	4 – 28 m
P3	F	11	143	41.7	III	SD	Bilateral solid AFOs	30 m	9 – 19 m
P4	M	13	158	38.5	III	SD	Bilateral solid AFOs and cane	67 m	9 – 19 m
P5	M	16	161	48.5	II	SH	None	93 m	4 – 28 m
P6	M	18	175	60.3	II	SH	None	22 m	4 – 28 m
P7	F	14	157	50	I	SH	None	31 m	4 – 28 m

^a^GMFCS: Gross Motor Function Classification System

^b^CP Type: spastic diplegia (SD); spastic hemiplegia (SH)

^c^Regular Assistive Device Use: assistive devices used on a typical basis by the participant; ankle foot orthosis (AFO)

^d^MCID: Minimum Clinically Important Difference, by GMFCS level [6]

## III. Discussion

The purpose of this study was to test the effect of providing instantly-adaptive ankle assistance from an untethered exoskeleton on maximal walking performance in children with CP. Our findings support the hypothesis that assistance from the device would significantly increase 6MWT performance when compared to unassisted walking after a short acclimation period, with average performance increasing beyond the MCID for this population [7]. While these exploratory findings should be interpreted with caution, our results contribute to a growing body of evidence that powered ankle assistance can improve walking performance in individuals with gait impairment, including from CP and stroke [8]. Ankle assistance did not affect walking efficiency, with no difference in Total Heart Beat Index (THBI) between Shod and Assisted conditions. This suggests that ankle assistance enabled participants to increase walking intensity, as indicated by increased distance, while maintaining the same energetic efficiency.

Performance on the Assisted 6MWT improved progressively across acclimation tests (Fig. 1(C)). This finding may be partially attributed to initial hesitation to externally-powered assistance during a maximal test that requires fast walking and sharp, 180 degree turns around a cone. The relatively rapid increase in performance (i.e., three days to pass the minimum clinically important difference) for users to trust and take advantage of exoskeleton assistance was an encouraging finding.

Overcoming technological challenges associated with providing reliable, beneficial assistance during fast, dynamic walking with sharp turns, this study represents a significant advancement over our prior work given that the 6MWT is a critical clinical metric of functional mobility and endurance capacity [9]. The present findings suggest that ankle exoskeleton assistance may be able to provide an impactful boost to free-living physical activity. We expect that simply walking with the device across the acclimation sessions likely transferred, in some capacity, to increased self-selected walking speed during walking without the device by cueing biomechanically favorable muscle activity coordination [10].

A notable limitation of this study was the lack of an initial Shod test on the first day before participants walked with assistance. Therefore, the results of this study are potentially artificially conservative for two reasons: (1) Shod was the only truly rested condition on test (third) day, and (2) neuromuscular priming resulting from the Assisted practice trials [10] may have artificially improved the Shod performance. Additionally, without a randomized controlled trial, it was not possible to eliminate any potential learning effect of completing the 6MWT multiple times from our exoskeleton acclimation results. Another limitation was that the walkway distance used in the study (20 m) was shorter than recommended (30 m). A shorter walkway, requiring more frequent turning, has been shown to result in a shorter 6MWT distance. Because the energetic benefit of the device is greater during walking than during turning, our results would likely have demonstrated even greater improvements from exoskeleton assistance if a longer walkway had been available. Lastly, given that Assisted 6MWT performance did not level off across visits (i.e., kept rising), future work should include a longer acclimation period to explore the upper limit of ankle exoskeleton assistance on this type of maximal walking test in CP.

## IV. Conclusion

Powered ankle assistance was able to significantly improve 6MWT performance in a small, functional cohort of individuals with CP after a short acclimation period without negatively affecting walking efficiency. We purposely compared assisted walking to walking without any assistive device even if a participant usually used one (e.g., ankle foot orthoses). While this comparison allowed us to isolate the effects of exoskeleton assistance on 6MWT performance, the benefits of powered assistance relative to common walking aids should be explored in future work.

## V. Materials and Methods

**Figure. 2.**
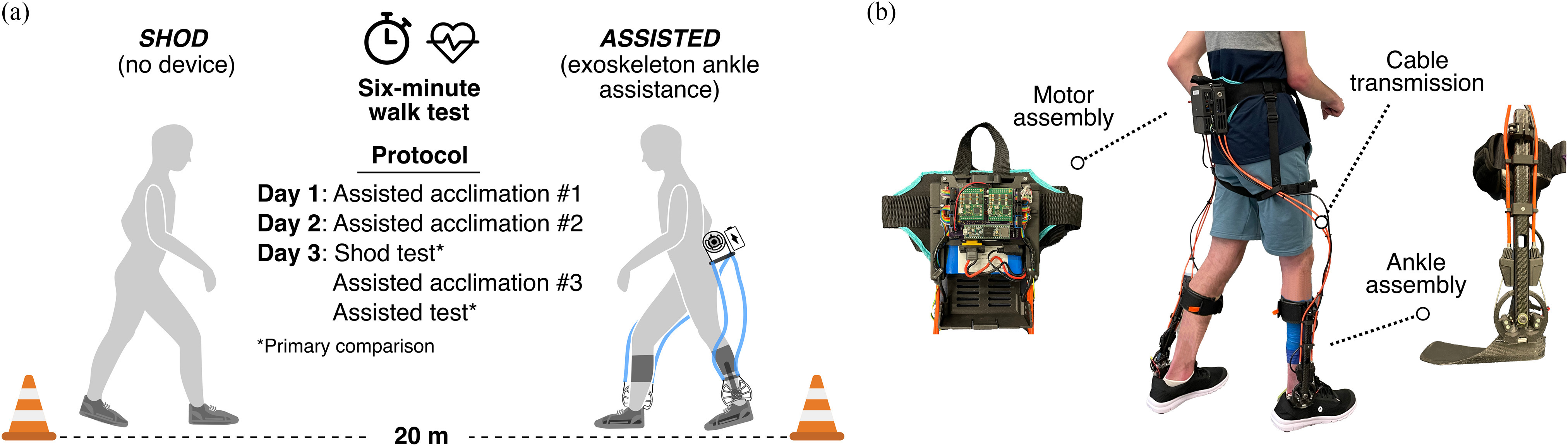
(a) Six-minute walk test protocol, consisting of three Assisted acclimation walks, one Shod test, and a final Assisted test; (b) Ankle exoskeleton device worn by all participants, which provided instantaneously adaptive assistance with ankle dorsi- and plantar flexion.

This three-day study (Fig. 2(A)) was approved by the Northern Arizona University Institutional Review Board (#986744), and prospectively registered at ClinicalTrials.gov (NCT04119063). Informed written consent was provided by the participant or his or her parent or legal guardian. Participants with a confirmed diagnosis of CP, ages 11 – 18 years, Gross Motor Function Classification System (GMFCS) levels I – III, and with the ability to complete a 6MWT with or without a walker were recruited for this three-day study.

Each participant was outfitted with a custom ankle exoskeleton device (Fig. 2(B)). Briefly, this untethered, battery- powered device consisted of a motor assembly worn at the waist, and bilateral ankle assemblies that provided stance-phase plantar flexor assistance proportional to each user's real-time, estimated ankle moment, seamlessly adjusting to turns and variable walking speed [11]; the peak nominal stance-phase torque setpoint was between 0.26 - 0.34 Nm/kg. As such, the stance-phase torque profile (shape and timing) was unique to each participant because it was governed by the ankle moment estimation scheme. During swing-phase, a constant level of dorsiflexor assistance (0.06 – 0.09 Nm/kg) was provided to ensure toe clearance for each participant. These levels were based on previous work that found energetically beneficial assistance ranges for individuals with CP [5], with slight adjustments for a participant's individual preference. The same gait-state transition thresholds were used for all participants. Even though our cohort was comprised of both diplegic and hemiplegic participants, bilateral assistance was provided to maximize the positive impact of the device on walking endurance by augmenting both limbs.

Participants completed three “acclimation” 6MWTs with the exoskeleton to acclimate to the maximal walking test with assistance (Assisted condition) on three separate days. Full acclimation to walking with an exoskeleton likely requires hours of repeated use over many days. To maximize the acclimation time in this study, we had participants complete a final acclimation walk on all three visits. On Day 3, participants started with an unassisted test (Shod condition without any assistive device), followed by two Assisted tests, each separated by 12 minutes. The “acclimated” final Assisted test was used for comparison to the Shod performance as the primary outcome. This order of testing was purposefully conservative to allow participants to complete their Shod test at their most rested state on Day 3, while also providing practice to the 6MWT procedure during the prior acclimation visits. As a secondary outcome, heart rate was recorded from a chest-mounted monitor (H10, Polar; Bethpage, NY, USA). Total Heart Beat Index (TBHI), a validated, minimally-disruptive measure of walking efficiency [12], was calculated as in (1):

\begin{equation*}
\frac{{{\rm{Total\ heart\ beats}}}}{{{\rm{Total\ distance\ traveled\ }}\left( {\rm{m}} \right)}} \tag{1}
\end{equation*}

All data sets were tested for normality using Shapiro-Wilk tests at the 5% significance levels. As the 6MWT data was not normally distributed, performance on this test was compared between the Shod and Assisted conditions using a Wilcoxon signed-rank test. The THBI data was normally distributed, so results were compared between conditions using a paired t-test. Significance was set at α < 0.05. Additionally, a linear regression was performed on 6MWT performance across the four Assisted condition tests to estimate the rate of acclimation.

## References

[1] H. K. Graham , “Cerebral palsy,” Nat. Rev. Dis. Prim., vol. 2, no. 1, Dec. 2016, Art. no. 15082.

[2] V. Dietz and W. Berger, “Cerebral palsy and muscle transformation,” Dev. Med. Child Neurol., vol. 37, no. 2, pp. 180–184, Nov. 2008. 10.1111/j.1469-8749.1995.tb11987.x 7851674

[3] B. C. Conner, N. M. Remec, C. M. Michaels, C. W. Wallace, E. Andrisevic, and Z. F. Lerner, “Relationship between ankle function and walking ability for children and young adults with cerebral palsy: A systematic review of deficits and targeted interventions,” Gait Posture, vol. 91, pp. 165–178, Jan. 2022. 34736095 10.1016/j.gaitpost.2021.10.024 PMC8671343

[4] D. L. Damiano, “Activity, activity, activity: Rethinking our physical therapy approach to cerebral palsy,” Phys. Ther., vol. 86, no. 11, pp. 1534–1540, Nov. 2006. 17094192 10.2522/ptj.20050397

[5] G. Orekhov, Y. Fang, J. Luque, and Z. F. Lerner, “Ankle exoskeleton assistance can improve over-ground walking economy in individuals with cerebral palsy,” IEEE Trans. Neural Syst. Rehabil. Eng., vol. 28, no. 2, pp. 461–467, Feb. 2020. 31940542 10.1109/TNSRE.2020.2965029 PMC7050636

[6] L. E. Mitchell, J. Ziviani, and R. N. Boyd, “Characteristics associated with physical activity among independently ambulant children and adolescents with unilateral cerebral palsy,” Dev. Med. Child Neurol., vol. 57, no. 2, pp. 167–174, Feb. 2015. 25146888 10.1111/dmcn.12560

[7] F. A. Storm , “Minimum clinically important difference of gross motor function and gait endurance in children with motor impairment: A comparison of distribution-based approaches,” Biomed Res. Int., vol. 2020, 2020. 10.1155/2020/2794036 PMC7246400 32509855

[8] L. N. Awad, P. Kudzia, D. A. Revi, T. D. Ellis, and C. J. Walsh, “Walking faster and farther with a soft robotic exosuit: Implications for post-stroke gait assistance and rehabilitation,” IEEE Open J. Eng. Med. Biol., vol. 1, pp. 108–115, Apr. 2020. 33748765 10.1109/OJEMB.2020.2984429 PMC7971412

[9] A. L. Fiss, L. Jeffries, K. Bjornson, L. Avery, S. Hanna, and S. W. McCoy, “Developmental trajectories and reference percentiles for the 6-minute walk test for children with cerebral palsy,” Pediatr. Phys. Ther., vol. 31, no. 1, pp. 51–59, Jan. 2019. 30557281 10.1097/PEP.0000000000000552

[10] Y. Fang, G. Orekhov, and Z. F. Lerner, “Adaptive ankle exoskeleton gait training demonstrates acute neuromuscular and spatiotemporal benefits for individuals with cerebral palsy: A pilot study,” Gait Posture, to be published, doi: 10.1016/j.gaitpost.2020.11.005. PMC8110598 33248858

[11] S. S. P. A. Bishe, T. Nguyen, Y. Fang, and Z. F. Lerner, “Adaptive ankle exoskeleton control: Validation across diverse walking conditions,” IEEE Trans. Med. Robot. Bionics, vol. 3, no. 3, pp. 801–812, Aug. 2021.

[12] V. L. Hood, M. H. Granat, D. J. Maxwell, and J. P. Hasler, “A new method of using heart rate to represent energy expenditure: The total heart beat index,” Arch. Phys. Med. Rehabil., vol. 83, no. 9, pp. 1266–1273, Sep. 2002. 12235607 10.1053/apmr.2002.34598

